# Supplementary material for: Researching COVID to enhance recovery (RECOVER) pregnancy study: Rationale, objectives and design
Source: PLoS One. 2023 Dec 21;18(12):e0285351. doi: 10.1371/journal.pone.0285351 (PMC10734909; doi:10.1371/journal.pone.0285351)
Supplement: S1 File — (DOCX) [file pone.0285351.s002.docx]

**RECOVER-Pregnancy Cohort Hubs and Sites**

| **Hub** | **Site** |
| --- | --- |
| NICHD Maternal-Fetal Medicine Units Network | University of Utah Health |
|  | Brown University |
|  | Christiana Care |
|  | Columbia University |
|  | Duke University |
|  | Intermountain Healthcare |
|  | Medical College of Wisconsin |
|  | Miami Valley Hospital |
|  | New York Presbyterian Queens |
|  | Northshore University Health System |
|  | Northwestern University |
|  | St. Peter's University Hospital |
|  | The George Washington University |
|  | The MetroHealth System |
|  | The Ohio State University |
|  | University Hospitals/Cleveland Medical Center |
|  | University of Alabama at Birmingham |
|  | University of Colorado |
|  | University of North Carolina at Chapel Hill |
|  | University of Pennsylvania |
|  | University of Pittsburgh |
|  | University of Texas at Medical Branch |
|  | University of Texas Health Science Center at Houston, Children's Memorial Hermann Hospital |
|  | WakeMed Health and Hospitals |
|  | Yale University |
| University of California San Francisco | University of California San Francisco |
