# Supplementary material for: Researching COVID to enhance recovery (RECOVER) pregnancy study: Rationale, objectives and design
Source: PLoS One. 2023 Dec 21;18(12):e0285351. doi: 10.1371/journal.pone.0285351 (PMC10734909; doi:10.1371/journal.pone.0285351)
Supplement: S2 File — (DOCX) [file pone.0285351.s003.docx]

# RECOVER-Pregnancy Consortium Members

## Study Group Lead Author

Torri D. Metz

[Torri.Metz@hsc.utah.edu](mailto:Torri.Metz@hsc.utah.edu)

Maternal-Fetal Medicine Unit University of Utah

University of Utah Health Services

*Torri D. Metz, Hub PI*

Jeanette P. Brown

Denise Lamb

Amanda L. Nelsen

Kim P. Phillips

Shannon M. Schlater

Jessica Sharma

McKaylee Smith

Amber Sowles

Jasmin Valencia Vazquez

Brown University

*Dwight J. Rouse, PI*

Donna M. Allard

Lisa M. Beati

Angelica M. DeMartino

Haley A. Lefebvre

Jane A. Milano

Emily S. Miller

ChristianaCare

*Matthew K. Hoffman, PI*

Carrie A. Kitto

Ashley Q. Vanneman

Columbia University

*Uma M. Reddy, PI*

Sabine Z. Bousleiman

Sara L. Eccheveri

Megan M. Loffredo

Andrea Perez

Rupa Ravi

Noelia M. Zork

Duke University

*Brenna L. Hughes, PI*

Jennifer W. Ferrara

Intermountain Healthcare

*M. Sean Esplin, PI*

Medical College of Wisconsin

*Anna Palatnik, PI*

Mariana Karasti

Eleanor Saffian

Miami Valley Hospital

*Samantha L. Weigand, PI*

Kathleen A. Fennig

Esther Kaye Snow

New York Presbyterian Queens

*Daniel W. Skupski, PI*

Rachel Pao

Honey Zaw

Northshore University Health System

*Beth A. Plunkett, PI*

Katharine Anglemire

Constandina Kapogiannis

Katherine Kearns

Quinlan Smith

Sunitha Suresh

Northwestern University

*Lynn M. Yee, PI*

Mercedes Brinson

Dequana D. Jones

Michelle A. Kominiarek

Gail L. Mallett

Trista Reynolds

Emily Y Williams

St. Peter's University Hospital

*Kristy T. S. Palomares, PI*

Imene Beche

Danielle Graziano-Carrete

Clara Perez

The George Washington University

*Rebecca G. Clifton, PI*

Katia J. Barrett

Celia M. Mullowney

Grecio J. Sandoval

Steven J. Weiner

The MetroHealth System

*Kelly S. Gibson, PI*

Wendy Dalton

Brittany Desantis

Parmjit Gill-Jones

Abigail Pierse

LuAnn Polito

Bonnie Rosolowski

Eugenia S. Sweet

The Ohio State University

*Maged M. Costantine, PI*

William A. Grobman

Anna B. C. Bartholomew

Barbara Cackovic

Cynthia Dembroski

Baylee Klopfenstein

Devra Mast

Kayla McDaniel

Melanie Paglione

Caitlin Rigsby

University Hospitals/Cleveland Medical Center

*David N. Hackney, PI*

University of Alabama at Birmingham

*Alan T. N. Tita, PI*

Donna J. Armstrong

Nicole P. Burrell

Brian M. Casey

Donna C. Dunn

Madison N. Mann

University of Colorado

*M. Camille Hoffman, PI*

Olivia Docter

Lauren M. Fischer

Jocelyn Phipers

University of North Carolina at Chapel Hill

*John M. Thorp, Jr. PI*

Kelly W. Clark

Molly A. Leatherland

Sally A. Timlin

University of Pennsylvania

*Samuel Parry, PI*

Anna Filipczak

Emily Long

Meaghan G. McCabe

Christina Pizzi

University of Pittsburgh

*Hyagriv N. Simhan, PI*

Francesca L. Facco

Frank C. Sciurba

John A. Vargo, IV

Jeanette E. Boyce

Sarah C. Hankle

Rachel L. Hines

Maura K. Hohn

Jill A. Tarr

University of Texas at Medical Branch

*Luis D. Pacheco, PI*

*George R. Saade, PI*

Jennifer A. Cornwell

Jennifer D. DeVolder

Amelia A. Nounes

Ashley E. Salazar

Lisa B. Thibodeaux

University of Texas Health Science Center at Houston, Children's Memorial Hermann Hospital

*Hector Mendez-Figueroa, PI*

Suneet P. Chauhan

Felecia Ortiz

Juanita Rugerio

Jenifer Treadway

WakeMed Health and Hospitals

*Carmen J. Beamon, PI*

Inez M. Dufresne

Chelsea A. Grinnan

Haliey M. Phillips

Yale University

*Christian M. Pettker, PI*

Lauren Perley

Linda Rink

University of California San Francisco

University of California San Francisco

*Vanessa Jacoby, Hub PI*

*Valerie Flaherman, Hub PI*

Nyat Araya

Cinthya Arellano-Mechor

Yangzom Basi

Ann Chang

Lauren Christopher

Isabel De La Torre

Soujanya Gade

Estefania Guerreros

Victoria Laleau

Susanne Martin Herz

Vanessa Monzon

Yulissa Oceguera Barragan

Michelle Rait

Marie Salem

Maria Tolentino

# RECOVER Patient, Caregiver, and Community Representatives

Sonseeahray (Ray) Adams^

Teresa Akintonwa

Leyna Aragon

Bryan Bander

Karyn Bishof

Frank Blancero

Gail Brooks^

Etienne Carignan

Megan Carmilani

Leah Castro-Baucom

Marta Cerda

Krista Coombs

Debra Copeland

Claudia Corchado

Hannah Davis

Felicia Davis Blakley

Marissa Diggs

Belinda Edwards

Heather Elizabeth Brown

Umar Favors

Whitney Fields^

Liza Fisher

Megan Fitzgerald

Alicia Gaffney

Margot Gage Witvliet

Roberto Garcia^

Tyler Gustafson

Nick Guthe

Yvonka Hall

Verna Holmes

Mady Hornig

Maxwell Hornig

Wendy Jefferson^

Christina Kim

Nancy Kochis

Kevin Kondo

Julie Lam

Fadwa Lawrence

Lydia Lerma

Rebecca Letts

Juan Lewis

Jacqui Lindsay

Silcia Lopez

Heather Marti

Thomas Martinez

Christine Maughan

Lisa McCorkell

Rebecca McGrath

Thomas Tony Minor

Charita Moore

Kian Nguyen

Lauren Nichols

Lisa O'Brien

Holly Olson

Aimee Peddie

Alice Perlowski

Elizabeth Phillips Lorenzo

Lisa Prentiss

Nadia Raytselis

Lidia Regino

Marjorie Roberts

Nitza Rochez

Megan Rockwell

Jacqueline Rutter

Nitza Rochez^

Elle Seibert

Anisha Sekar

Chimere Smith^

Lauren Stiles

Brittany Taylor

Emily Taylor

Julie Thompson

Stephen Trapp^

Stephen Valdiva

Hyatt Vincent

Ann Wallace

Rochelle Wilensky

Melissa Williams

Neely Williams

Kay Williams Dawson

Andrew Wylam

Mike Zissis

# RECOVER Cores

Administrative Coordinating Center at Research Triangle Institute International

*Lisa Newman, PI*

Quinn Barnette

Patricia Ceger

Mike Enger

Katie Fain

Tonya Farris

Sean Hanlon

David Hines

Kevin Jordan

Beth Linas

Meisha Mandal

Susan Nance

Lisa Newman

Claire Quiner

Rita Sembajwe

Gwendolyn Shaw

Vanessa Thornburg

Kendall Tosco

Clinical Science Core at NYU Langone Health

*Rachel Gross, PI*

*Judith Hochman, PI*

*Leora Horwitz, PI*

*Stuart Katz, PI*

*Andrea Troxel, PI*

Lenard Adler

Preciosa Akinbo

Ramona Almenana

Malate Aschalew^

Lara Balick^

Ola Bello

Sultana Bhuiyan

Nina Blachman

Ryan Branski

Jasmine Briscoe

Shari Brosnahan

Elliott Bueler

Yvette Burgos

Nina Caplin

Dominique Chaplin

Yu Chen

Shen Cheng

Myong (Jess) Choi

Alicia Chung

Richard Church

Stanley Cobos

Nakia Croft

Angelique Cruz Irving

Phoebe Del Boccio

Iván Díaz

Jasmin Divers

Vishal Doshi

Benard Dreyer

Samantha Ebel

Shari Esquenazi-Karonika

Arline Faustin

Elias Febres

Jeffrey Fine

Sandra Fink

Catherine Freeland

Jennifer Frontera

Richard Gallagher

Alejandra Gonzalez-Duarte

Denise Hasson

Sophia Hill

Jennifer Hossain^

Shahidul Islam

Stephen Johnson

Neha Kansal

Rachel Kenney

Tammy Kershner^

Deepshikha Kewlani

Judy Kwak^

Michelle F. Lamendola-Essel

Sarah Laury^

Gregory Laynor

Lei Lei^

Terry Leon

Janelle Linton

Max Logan

Nadia Malik

Lia Mamistvalova

Hannah Mandel

Gabrielle Maranga

Aprajita Mattoo

Tony Mei

Alan Mendelsohn

Emmanuelle Mercier

Patricio Millar Vernetti

Marc Miller

Maika Mitchell

Andre Moreira

Praveen C. Mudumbi

Erica Nahin

Nandini Nair

Joseph Nekulak

Kellie Owens

Brendan Parent

Nandan Patibandla

Peter Petrov

Radu Postelnicu

Francesca Pratt

Isabelle Randall

Priyatha Rao

Amy Rapkiewicz

J.R. Rizzo

Johana Rosas

Chelsea Rose

Christina Saint Jean

Michelle Santacatterina

Binita Shah

Aasma Shaukat

Naomi Simon

Aylin Simsir

Miranda Stinson

Wenfei Tang

Vasishta Tatapudi

Sujata Thawani

Mary Thomas

Lorna Thorpe

MeeLee Tom

Ethan Treiha

Jennifer Truong

Mmekom Udosen

Carlos Valencia^

Jessica Velazquez-Perez

Crystal Vidal

Anand Viswanathan

Amy Willerford^

Natasha Williams^

Crystal Wong

Marion J. Wood

Shannon Wuller

Shonna Yin

Chloe Young

Jonah Zaretsky

Susanna Zavlunova

Data Repository Core at Massachusetts General Hospital

*Andrea Foulkes, PI*

*Elizabeth Karlson, PI*

*Shawn Murphy, PI*

Shreya Ahirwar

Shifa Ahmed

Rachel Atchley

Marie-Abèle C. Bind

William Bonaventura

Natalie Boutin

Tingyi Cao

Victor Castro

James Chan

Lori B. Chibnik

Maria Fayad

Vivian Gainer

Randy Gollub

Karl Helmer

Daniel Kaufman

Aparna Krishnamoorthy

Katherine P. Liao

Megan Martel

Jonathan Monteiro

Richard E. Morse

Amber Nguyen

Henry H. Paik

Deepti Pant

Dustin J. Rabideau

Harrison T. Reeder

Katherine Schlepphorst

Carolin Schulte

Daniel J. Shinnick

Caitlin Selvaggi

Lynn Simpson

Mary L. St. Jean

Mansi Thakrar

Tanayott Thaweethai

Nich Wattansain

Griffin Weber

PASC Biorepository Core at Mayo Clinic

*Mine Cicek, PI*

Nancy Chang

Evan Ellingworth

Jordan Weyer

Jennifer Wheeler

Samantha Wirkus

Nicole Zahnle

Reading Centers

Natalie Boutin

Shari Brosnahan

Xander Cerretani

Randy Gollub

Rachel Gross

Karl Helmer

Michelle Lamendola-Essel

Gabrielle Maranga

Caroline Moorehouse

Jennifer Payne

# RECOVER Committees and Task Forces

Executive

Hugh Auchincloss

Diana Bianchi

Joe Breen

Patti Brennan

Jeffrey Burns

Nakela Cook

Felicia Davis Blakley

Betty Diamond

Mitchell S.V. Elkind

Lee Fleisher

Andrea Foulkes

Gary Gibbons

Laurie Gutmann

Michael Iademarco

Stuart Katz

Walter Koroshetz

Eldrin F. Lewis

Peter Marks

Hilary Marston

Mitchell Miglis

Lisa Newman

Tracy Nolen

Carlos A. Pardo-Villamizar

Amy Patterson

Sam Posner

Wendy S. Post

Serena Spudich

Clinton Wright

Heather Yates

Kanecia Zimmerman

Steering

Gabriel Anaya

Audie Atienza

Charles Bailey

R. Graham Barr

Lisa Berdan

Andra Blomkalns

Melissa Bondy

Hassan Brim

Jeffrey Burns

Alexander Charney

Benjamin Chen

Mine Cicek

John Crary

Dawood Darbar

Kathi Diviak

Ray Ebert

Jamie Elifritz

Robert L. Ferrer

Josh Fessel

Aloke Finn

Thomas Flotte

Paul Fontelo

Andrea Foulkes

Emily Gallagher

Maria Gennaro

Mary Groesch

Rachel Gross

Melissa Haendel

James Heath

Rachel Hess

Stephen Hewitt

Sally Hodder

Carol Horowitz

Leora Horwitz

Vanessa Jacoby

Sarah Jolley

Suzanne Judd

Bill Kapogiannis

Elizabeth Karlson

Barbara Karp

Stuart Katz

Rainu Kaushal

Lawrence Kleinman

Jerry Krishnan

Sweta Ladwa

Craig Lefebvre

Lei Lei^

Emily Levitan

Bruce Levy

Daniel Liu

Jeffrey Martin

Grace McComsey

Robin J. Mermelstein

Torri Metz

Lucio Miele

Sindhu Mohandas

Janet Mullington

Shawn Murphy

Jane Newburger

Lisa Newman

Margaret Ochocinska

Igho Ofotokun

Princess Ogbogu

Michelle Olive

Sairam Parthasarathy

Amy Patterson

Thomas Patterson

Gail Pearson

Priscilla Pemu

James Porterfield

Antonello Punturieri

R. Ross Reichard

Jane Reusch

Kyung Rhee

Kathleen Rodgers

Juan Salazar

Amy Salisbury

Lumy Sawaki-Adams

Lisa Schwartz-Longacre

Sudha Seshadri

Howard Sesso

Eyal Shemesh

Allan Shipp

Upinder Singh

Jessica Snowden

Serena Spudich

Melissa Stockwell

James Stone

Jun Sun

Mehul Suthar

David Systrom

Brittany Taylor

Stephen Thibodeau

Andrea Troxel

PJ Utz

Tiffany Walker

David Warburton

Gail Weinmann

Neely Williams

Dana Wolff-Hughes

John Wood

Adjudication

Khamal Anglin

Emilia Bagiella

Ryan Branski

Rodica Busui

Marissa Diggs

Vivian Gainer

Sunanda Gaur

Linda Geng

Sarah Jolley

Sarah Laury^

Jai Marathe

Lisa McCorkell

Jarrod Mosier

Binita Shah

Dimpy Shah

Tiffany Walker

Peter Whitesell

Ancillary Studies

Hassan Ashktorab

Jeannette Beasley

Karyn Bishof

Yu Chen

Lori Chibnik

Dani Dumitriu

Jennifer Frontera

Paul Goepfert

Sylvie Goldman

Stephen Hewitt

Matt Huentelman

Barbara Karp

Jerry Krishnan

Sarah Laury^

Bruce Levy

Janko Nikolich-Zugich

Margaret Ochocinska

Laura Pace

Alice Perlowski

W. Brian Reeves

Juan Salazar

Lumy Sawaki-Adams

Sujata Thawani

Hannah Valantine

Drenna Waldrop

Dana Wolff-Hughes

Cardiopulmonary

Natasha Altman

Tell Bennett

Soham DasGupta

Marissa Edminston

Aloke Finn

Tyler Gustafson

Francois Haddad

Jennifer Hossain^

Priscilla Hsue

Pavitra Kotini-Shah

Sankaran Krishnan

Anu Lala-Trindade

Simon Lee

Alem Mehari

Patricio Millar Vernetti

Andre L. Moreira

Anoop Nambiar

Robert Padera

Gail Pearson

Dhaval Raval

Franz Rischard

Erika Rosenzweig

Barbara Sampson

Frank Sciurba

Jackie Szmuszkovicz

Julie Thompson

Dongngan Truong

Viola Vaccarino

George Washko

John Wood

Commonalities with Other Post-viral Syndromes

Hector Bonilla

Christine Capone

Sekai Chideya-Chihota

Dane Cook

Monica Gutierrez

Rohan Hazra

Leonard Jason

Phillip Joseph

Dan Kelly

Joyce Lee-Ianotti

Vincent Marconi

Joshua Milner

Benjamin Natelson

Lisa O'Brien

Carlos Oliveira

James Porterfield

Claire Quiner

Zaki Sherif

Nora Singer

Inderjit Singh

David Systrom

C. Sabrina Tan

Emily Taylor

Vanessa Thornburg

Suzanne Vernon

Core Operations Group

Quinn Barnette

Frank Blancero

Mine Cicek

Lauren Decker

Jasmin Divers

Ray Ebert

Tonya Farris

Valerie Flaherman

Thomas Flotte

Andrea Foulkes

Rachel Gross

Sally Hodder

Leora Horwitz

Elizabeth Karlson

Stuart Katz

Craig Lefebvre

Lei Lei^

Shawn Murphy

Lisa Newman

Michelle Olive^

Tony Punturieri

Lisa Schwartz Longacre

Upinder Singh

Stephen Thibodeau

Andrea Troxel

David Warburton

Jordan Weyer

Health Equity/ Patient Reported Outcomes / Community Engagement

Brett Anderson^

Sujata Bardhan

Leah Castro-Baucom

Deena Chisolm

Alicia Chung

Claudia Corchado

Casey Daniel

Walter Dehority

Gniesha Dinwiddie

Candace H. Feldman

Lisa Goldman Rosas

Carol Horowitz

Janice John

Gelise Little John Thomas

Keila Lopez

Karen Lutrick

Carina Marquez

Shelly McDonald Pinkett

Lidia Regino

Kim Rhoads

Sarah Stewart de Ramirez

Joel Tsevat

Carlos Valencia^

Nita Vangeepuram

Anita Walden

Zanthia Wiley

Neely Williams

Shonna Yin

Immunology & Hematology

Galit Alter

Hulya Bukulmez

Jiang Chao

Ai-Ris Collier

Karen H. Costenbader

Betty Diamond

Rao Divi

Nahed El Kassar

Nathan Erdmann

Frances Eun-Hyung Lee

Alicia Gaffney

Sacha Gnjatic

Jason Goldman

Jim Heath

Jennifer Hossain^

Hye-Sook Kim

Ellen Kraig

Timothy Lewis

Joy Liu

Gabrielle Maranga

Aprajita Mattoo

Joshua Milner

Sindhu Mohandas

Janko Nikolich-Zugich

Princess Ogbogu

Michael Peluso

Bellur Prabhakar

Jay Raval

Marian Sullivan

Paul Thuluvath

PJ Utz

Sidney Whiteheart

Integrative Physiology

Nina Caplin

Dawood Darbar

Katie Fain

Aloke Finn

Thomas Flotte

David Goldstein

Meredith Hay

Ellie Hirshberg

Barbara Karp

Dean Kellogg Jr.

Rebecca Letts

Meisha Mandal

Janet Mullington

Michael Portman

David Putrino

Jacqueline Rutter

Lumy Sawaki-Adams

Joel Trinity

John Wood

Roham Zamanian

Interventions

Judy Aberg

Farshad Aduli

Andrew Atz

Lawrence Baizer

Hector Bonilla

Patricia Ceger

Maged Costantine

Amelia Deitchman

Kelly Filipski

Jeffrey Fine

Liza Fisher

Paul Fontelo

Vilma Gabbay

Jason Goldman

Mi Hillefors

Verna Holmes

Arthur Kim

Grace McComsey

Gregory Mertz

Sindhu Mohandas

Margaret Ochinska

Thomas Patterson

Michael Peluso

John Quigley

Kathleen Rodgers

Nadine Rouphael

Gwen Shaw

Bob Tamburro

David Williams

William Zempsky

Mechanistic Pathways

Christian Bime

Steven Bradfute

Benjamin Chen

Tom Connors

Krista Coombs

Glenn Fishman

Maria Gennaro

Christian Gomez

Timothy Henrich

David Hines

Prasanna Jagganathan

Judith James

Kevin Jordan

Boris Juelg

Christina Kim

Jining Lu

Sindhu Mohandas

Michael Portman

Erin Quinlan

Brian Reeves

Jalees Rehman

Ignacio Sanz

Zaki Sherif

Guofei Zhou

Metabolic Disorders

Irina Buhimschi

Floyd Chilton

Ralph DeFronzo

Emily Gallagher

Jennifer Hossain^

Wendy Jefferson

Mandana Khalili

Angesom Kibreab

Tracey McLaughlin

Nandini Nair

Venkat Narayan

Elizabeth Phillips Lorenzo

Jane Reusch

Ivonne Schulman

Aasma Shaukat

Deborah Wexler

Jonah Zaretsky

Microbiology

William Alexander

Leyna Aragon

Ami Bhatt

Hassan Brim

Shari Brosnahan

John Coffin

Adolfo Garcia-Sastre

Maria Gennaro

Joerg Graf

Timothy Henrich

Nahed Ismail

Paul Keim

Hye-Sook Kim

Jeanne Marrazzo

Rebecca McGrath

Sindhu Mohandas

Christopher Montgomery

Radu Postelincu

Ryan Ranallo

Kentner Singleton

Adam Spivak

Mehul Suthar

Mary Thomas

Guangming Zhong

National Community Engagement Group

Teresa Akintonwa

Jasmine Briscoe

Heather Elizabeth Brown

Megan Carmilani

Marta Cerda

Debra Copeland

Felicia Davis Blakely

Yvonka Hall

Kevin Kondo

Fadwa Lawrence

Lydia Lerma

Jacqui Lindsay

Christine Maughan

Thomas Minor

Marjorie Roberts

Nitza Rochez

Brittany Taylor

Hyatt Vincent

Heather Yates

Neuropsychiatric

John Andrefsky

Bryan Bander

Douglas Bremner

Michael Carrithers

Melissa Cortez

Richard Gallagher

Alejandra Gonzalez Duarte

Joanna Hellmuth

Tammy Kershner^

Mansoor Malik

Shawn Murphy

Ganesh Murthy

Sharon H. O'Neil

Lisa Prentiss

Caitlin Rollins

Jonathan Rosand

Scott Russo

Amy Salisbury

Alan Seifert

Sudha Seshadri

Eyal Shemesh

Wendy Silver

Naomi Simon

Leanne Williams

Omics

Masanori Aikawa

Hassan Ashktorab

Noam Beckmann

Jasmin Divers

Mike Enger

Stephen Erickson

Joaquin Espinosa

Xiaowu Gai

Stephen Hewitt

Benjamin Horne

Paul Keim

Jessica Lasky-Su

Cheryl Maier

Meisha Mandal

Lucio Miele

Emmanuel Mongodin

Lauren Nichols

Nadia Roan

Mark Russell

George Saade

Kumar Sharma

Stephanie Shiau

Jun Sun

Stephen Thibodeau

Sam Yang

Participant Experience

Nina Blachman

Natalie Boutin

Phoebe Burton

Marina Catallozzi

Cheryl Clark

Beth Dworetzky

Belinda Edwards

Robert L. Ferrer

Catherine Freeland

Beatrice Huang

Suzanne Judd

Sarah Laury^

Hugh Musick

Divya Pathak

Gail Pearson

Kristen Pogreba-Brown

Hengameh Raissy

Lynne Richardson

Russell Rothman

Laura Wagner

Ann Wallace

Population Science

Stephanie Archer

Paul Barach

Melissa Bondy

Victor Castro

Mine Cicek

Joanne Elena

Kacey Ernst

Josh Fessel

Dan Fort

Brian Hendricks

Bertha Hidalgo

Cory Hussain

Carmen Isasi

Dan Kelly

Adeyinka Laiyemo

Margaret Lanca

Juan Lewis

Beth Linas

Heidi May

Kimberly McHugh

Naoko Muramatsu

Girish Nadkarni

Susan Nance

Kian Nguyen

Priscilla Pemu

Lisa Postow

Suchitra Rao

Dimpy Shah

Sidd Shenoy

Kendall Tosco

Dana Wolff-Hughes

Presentations and Publications Oversight

Ingrid Bassett

Diana Berrent

Andra Blomkalns

Hassan Brim

Rebecca Clifton

Nathan Erdmann

Kristine Erlandson

Valerie Flaherman

Margot Gage-Witvliet

Mark Goldberg

Edmond Kabagembe

Tammy Kershner^

Patricia Kinser

Jonathan Klein

Gregory Laynor

Grace McComsey

Brian McCrindle

Julie McMurry

Girish Nadkarni

Priscilla Pemu

Dustin Rabideau

Erika Rosenzweig

Sarah Stewart de Ramirez

David Warburton

QA/QC Data Integrity

Charlie Bailey

James Chan

Nancy Chang

Mine Cicek

Hannah Davis

Kathi Diviak

Dan Fort

Jennifer Gander

Janos Hajagos

Kellie Hawkins

Shahidul Islam

Dan Kelly

Tammy Kershner^

Patricia Kovatch

Simon Li

Daniel Liu

Holden Maecker

Faran Mahmood

Emily Pfaff

Anisha Sekar

Zaki Sherif

Vignesh Subbian

Mansi Thakrar

Anand Viswanathan

Jennifer Wheeler

Meredith Zozus

Study Design

Yvette Burgos

Chris Chute

Elizabeth Karlson

Barbara Karp

Adeyinka Laiyemo

Emily Levitan

Gabrielle Maranga

Gailen Marshall

Jeffrey Martin

Robin Mermelstein

Torri Metz

Igho Ofotokun

Sairam Parthasarathy

Lumy Sawaki-Adams

Mary Beth Scholand

Howard Sesso

Nora Singer

Lauren Stiles

Barbara Taylor

Juan Wisnivesky

*^discontinued effort on RECOVER*
