# Supplementary material for: Researching COVID to enhance recovery (RECOVER) pregnancy study: Rationale, objectives and design
Source: PLoS One. 2023 Dec 21;18(12):e0285351. doi: 10.1371/journal.pone.0285351 (PMC10734909; doi:10.1371/journal.pone.0285351)
Supplement: Attachment — Submitted filename: Response to Reviewers.docx [file pone.0285351.s004.docx]

Thank you for the opportunity to revise our manuscript. Please see below for point-by-point responses to the comments from the reviewers and editors.

Journal Requirements:

When submitting your revision, we need you to address these additional requirements.

1. Please ensure that your manuscript meets PLOS ONE's style requirements, including those for file naming. The PLOS ONE style templates can be found at

[https://journals](https://journals/).plos.org/plosone/s/file?id=wjVg/PLOSOne_formatting_sample_main_body.pdf and 
<https://journals.plos.org/plosone/s/file?id=ba62/PLOSOne_formatting_sample_title_authors_affiliations.pdf>

**Response:** We confirm that we have formatted the manuscript according to journal style requirements.

2. Thank you for stating the following in the Competing Interests section:

"I have read the journal's policy and the authors of this manuscript have the following competing interests:
Dr. Metz reports personal fees from Pfizer for her role as a medical consultant for a SARS-CoV-2 vaccination in pregnancy study, grants from Pfizer for role as a site PI for SARS-CoV-2 vaccination in pregnancy study, grants from Pfizer for role as a site PI for RSV vaccination in pregnancy study, and grants from Gestvision for role as a site PI for a preeclampsia study outside the submitted work. Dr. Horwitz reported serving as a member of the National Academy of Medicine Committee on the Long-Term Health Effects Stemming from COVID-19 and Implications for the Social Security Administration. Dr. Costantine reported receiving grant support for work not related to this paper from Baxter International and Siemens Healthcare and personal consulting fees not related to this paper from Progenity and Siemens Healthcare."

We note that you received funding from a commercial source: Pfizer, Gestvision, Baxter International, Progenity and Siemens Healthcare

Please provide an amended Competing Interests Statement that explicitly states this commercial funder, along with any other relevant declarations relating to employment, consultancy, patents, products in development, marketed products, etc.

Within this Competing Interests Statement, please confirm that this does not alter your adherence to all PLOS ONE policies on sharing data and materials by including the following statement: "This does not alter our adherence to PLOS ONE policies on sharing data and materials.” (as detailed online in our guide for authors [http://journals](http://journals/).plos.org/plosone/s/competing-interests).  If there are restrictions on sharing of data and/or materials, please state these.

Please note that we cannot proceed with consideration of your article until this information has been declared.

Please include your amended Competing Interests Statement within your cover letter. We will change the online submission form on your behalf.

**Response:** We have provided our amended Competing Interests Statement within our cover letter as requested.

3. PLOS requires an ORCID iD for the corresponding author in Editorial Manager on papers submitted after December 6th, 2016. Please ensure that you have an ORCID iD and that it is validated in Editorial Manager. To do this, go to ‘Update my Information’ (in the upper left-hand corner of the main menu), and click on the Fetch/Validate link next to the ORCID field. This will take you to the ORCID site and allow you to create a new iD or authenticate a pre-existing iD in Editorial Manager. Please see the following video for instructions on linking an ORCID iD to your Editorial Manager account: <https://www.youtube.com/watch?v=_xcclfuvtxQ>

**Response:** Dr. Metz has linked her ORCID iD in the Editorial Manager system.

4. Please amend either the title on the online submission form (via Edit Submission) or the title in the manuscript so that they are identical.

**Response:** Title in the online submission form has been corrected.

5. One of the noted authors is a group or consortium: The RECOVER Initiative

In addition to naming the author group, please list the individual authors and affiliations within this group in the acknowledgments section of your manuscript. Please also indicate clearly a lead author for this group along with a contact email address.

**Response:** All individual consortial authors are listed in a supplemental appendix.

6. Please include your full ethics statement in the ‘Methods’ section of your manuscript file. In your statement, please include the full name of the IRB or ethics committee who approved or waived your study, as well as whether or not you obtained informed written or verbal consent. If consent was waived for your study, please include this information in your statement as well.

**Response:** The RECOVER pregnancy cohort study was approved by the New York University IRB. This was already included in the Methods section of the manuscript on p. 24-25.

All RECOVER activities are approved by a single institutional review board (IRB), which is the New York University IRB.

Written informed consent will be obtained for all participants by research personnel at participating RECOVER-Pregnancy sites.

7. Please include captions for your Supporting Information files at the end of your manuscript, and update any in-text citations to match accordingly. Please see our Supporting Information guidelines for more information: [http://journals](http://journals/).plos.org/plosone/s/supporting-information.

**Response:** Captions are now provided at the end of the manuscript.

8. We note that the original protocol that you have uploaded as a Supporting Information file contains an institutional logo. As this logo is likely copyrighted, we ask that you please remove it from this file and upload an updated version upon resubmission.

**Response:** The institutional logo has not been removed. The rationale for this is that PLOS One has previously published the RECOVER Adult Study Design Manuscript with the study protocol containing the NYU institutional logo. The RECOVER Pregnancy Cohort uses the same base protocol. Thus, this recommendation is not consistent with prior guidance from this journal.

9. We note that the original protocol file you uploaded contains a confidentiality notice indicating that the protocol may not be shared publicly or be published. Please note, however, that the PLOS Editorial Policy requires that the original protocol be published alongside your manuscript in the event of acceptance. Please note that should your paper be accepted, all content including the protocol will be published under the Creative Commons Attribution (CC BY) 4.0 license, which means that it will be freely available online, and any third party is permitted to access, download, copy, distribute, and use these materials in any way, even commercially, with proper attribution.

Therefore, we ask that you please seek permission from the study sponsor or body imposing the restriction on sharing this document to publish this protocol under CC BY 4.0 if your work is accepted. We kindly ask that you upload a formal statement signed by an institutional representative clarifying whether you will be able to comply with this policy. Additionally, please upload a clean copy of the protocol with the confidentiality notice (and any copyrighted institutional logos or signatures) removed.

**Response:** A formal statement has been uploaded from the institutional official indicating that the study protocol can be published. Please see note above. This is the same study protocol for the RECOVER Adult study, which was previously published by this journal.

10. Please review your reference list to ensure that it is complete and correct. If you have cited papers that have been retracted, please include the rationale for doing so in the manuscript text, or remove these references and replace them with relevant current references. Any changes to the reference list should be mentioned in the rebuttal letter that accompanies your revised manuscript. If you need to cite a retracted article, indicate the article’s retracted status in the References list and also include a citation and full reference for the retraction notice.

**Response:** References have been reviewed and are correct.

[Note: HTML markup is below. Please do not edit.]

**Reviewers' comments:**

Reviewer's Responses to Questions

**Comments to the Author**

1. Does the manuscript provide a valid rationale for the proposed study, with clearly identified and justified research questions?

The research question outlined is expected to address a valid academic problem or topic and contribute to the base of knowledge in the field.

Reviewer #1: Yes

Reviewer #2: Yes

Reviewer #3: Yes

2. Is the protocol technically sound and planned in a manner that will lead to a meaningful outcome and allow testing the stated hypotheses?

The manuscript should describe the methods in sufficient detail to prevent undisclosed flexibility in the experimental procedure or analysis pipeline, including sufficient outcome-neutral conditions (e.g. necessary controls, absence of floor or ceiling effects) to test the proposed hypotheses and a statistical power analysis where applicable. As there may be aspects of the methodology and analysis which can only be refined once the work is undertaken, authors should outline potential assumptions and explicitly describe what aspects of the proposed analyses, if any, are exploratory.

Reviewer #1: Yes

Reviewer #2: Yes

Reviewer #3: Yes

3. Is the methodology feasible and described in sufficient detail to allow the work to be replicable?


Descriptions of methods and materials in the protocol should be reported in sufficient detail for another researcher to reproduce all experiments and analyses. The protocol should describe the appropriate controls, sample size calculations, and replication needed to ensure that the data are robust and reproducible.

Reviewer #1: Yes

Reviewer #2: Yes

Reviewer #3: Yes

4. Have the authors described where all data underlying the findings will be made available when the study is complete?

The [PLOS Data policy](https://journals.plos.org/plosone/s/materials-and-software-sharing) requires authors to make all data underlying the findings described in their manuscript fully available without restriction, with rare exception, at the time of publication. The data should be provided as part of the manuscript or its supporting information, or deposited to a public repository. For example, in addition to summary statistics, the data points behind means, medians and variance measures should be available. If there are restrictions on publicly sharing data—e.g. participant privacy or use of data from a third party—those must be specified.

Reviewer #1: No

Reviewer #2: Yes

Reviewer #3: Yes

5. Is the manuscript presented in an intelligible fashion and written in standard English?

PLOS ONE does not copyedit accepted manuscripts, so the language in submitted articles must be clear, correct, and unambiguous. Any typographical or grammatical errors should be corrected at revision, so please note any specific errors here.

Reviewer #1: Yes

Reviewer #2: Yes

Reviewer #3: Yes

6. Review Comments to the Author

Please use the space provided to explain your answers to the questions above and, if applicable, provide comments about issues authors must address before this protocol can be accepted for publication. You may also include additional comments for the author, including concerns about research or publication ethics.

You may also provide optional suggestions and comments to authors that they might find helpful in planning their study.


(Please upload your review as an attachment if it exceeds 20,000 characters)

**Reviewer #1:** In this study protocol, RECOVER-Pregnancy will investigate whether having SARS-CoV-2 during pregnancy modifies the risk factors, prevalence, and phenotype of post-cute sequelae of SARS-CoV-2. Both prospective and retrospective cohorts will be enrolled. Adults with pregnancy during the COVID-19 pandemic and their offspring exposed and unexposed in utero will be included. The expected total sample size is 2,300 (1867 infected / 433 uninfected).

Minor revision:
Identify the software that will be used for the statistical analysis.

**Response:** R software will be used for statistical analyses. This information has been added to the manuscript.

“Statistical analyses will be performed using R software.”

**Reviewer #2:** The authors propose a large scale, multicentre cohort follow up study of pregnant individuals (and their offspring) exposed to Sars-CoV-2 during pregnancy. This proposal is closely related to the RECOVER-Adult and Paediatric cohorts, utilising the same methodological approaches with important modifications to account for pregnancy.
This is an important study, which is appropriately designed. The tiered approach allows for the opportunity to obtain more detailed information and samples from a much smaller cohort, thereby helping to minimise participant burden.
The authors appropriately address the limitations of the design (eg limited ability to gain early assessments from those exposed to the earlier COVID variants) and justify the pregnancy specific modifications.
The team of researchers have significant clinical and research experience to undertake this work.
All key components of the protocol appear to be included in this manuscript. Appropriate management and quality assurance structures appear to be in place. IRB approvals are in place.

**Response:** Thank you. No revisions needed in response to this review.

**Reviewer #3:** Thank you for the opportunity to review this submission. The manuscript is well written and the protocol is describe in sufficient detail. I have a few comments for the authors consideration:

1) Line 279: please clarify if men (male sex assigned at birth) are eligible as part of the non-pregnant adult participants . Similarly, whether women who are outside of "traditional" child bearing ages are eligible. With a significantly smaller unexposed compared to exposed group this could raise concerns about how exchangable your exposed and unexposed study samples really are. Based on the sample size presented just prior to this, it may just be the language that needs to be fixed for clarity (i.e., assuming 25% of non-pregnant adult participants are female..).

**Response:** We have clarified that comparisons are planned with non-pregnant female participants in the RECOVER-Adult Cohort on p. 8, 9, 10 and 26.

Thus, it remains unknown if there is a differential likelihood of PASC among individuals who contract SARS-CoV-2 during pregnancy compared with non-pregnant reproductive age females.

We hypothesize that the incidence of persistent long-term sequelae among pregnant people will vary from that of non-pregnant reproductive age females given the unique changes in physiology, hormone levels, and inflammatory milieu in pregnancy.

…and (3) evaluating if contracting SARS-CoV-2 in pregnancy is associated with a differential risk of developing PASC compared with non-pregnant reproductive age females.

Differences in the prevalence or symptoms of PASC among those who acquire SARS-CoV-2 in pregnancy compared with non-pregnant reproductive age females could help with determination of the underlying pathophysiology of PASC.

2) following the above point, it looks like in the sample size calculations you are including unexposed from the recover-adult cohort for a pregnant to non-pregnant comparison and it's still a little confusing as to whether this will be restricted by sex at birth to females only.

**Response:** Text was further modified for clarity on p. 13. There is also a statement on p.24 clarifying that comparisons will be made to non-pregnant reproductive age females.

Assuming 25% of non-pregnant adult participants are female and of reproductive age, the minimum detectable PASC risk difference between SARS-CoV-2 infected reproductive age female participants with and without active pregnancy at infection is 4.7% (8.9% in a 25% subgroup).

Logistic regression analyses will be conducted to investigate associations between cumulative incidence of PASC and clinical factors including vaccination status, demographics, and social determinants of health among participants with infection during pregnancy, as well as comparing those infected during pregnancy with infected non-pregnant reproductive age females.

3) Statistical analysis section - it looks like you powered everything for a risk difference but most of the stats section related to odds ratios and logistic regression. Could be useful to describe those methods in more detail, right now it reads more as an added rather than primary analysis.

**Response:** The sample size and power calculations on pp. 13-15 report minimum detectable differences in PASC risk due to their ease of interpretation, though as the reviewer notes our primary analyses of PASC risk are planned as logistic regression models which estimate odds ratios. This choice does not affect the design of the study, as minimum detectable risk differences can be equivalently expressed as minimum detectable odds ratios to correspond with the planned logistic regression analyses. For completeness, we have updated Table 1 to report both sets of minimum detectable effects.

Minor comments:
1) Recommend using the term unexposed instead of controls as you are neither randomizing nor performing a case-control study.

**Response:** Thank you for this suggestion. All instances of “control” were changed to “unexposed” or “uninfected participants”.

2) Could be useful to discuss briefly how you will handle vaccination status in this.

**Response:** Vaccination status will be considered when appropriate in analyses related to the development of PASC. This covariate for modeling is now specifically mentioned on p. 24.

Logistic regression analyses will be conducted to investigate associations between cumulative incidence of PASC and clinical factors including vaccination status, demographics, and social determinants of health among participants with infection during pregnancy, as well as comparing those infected during pregnancy with infected non-pregnant reproductive age females.

7. PLOS authors have the option to publish the peer review history of their article ([what does this mean?](https://journals.plos.org/plosone/s/editorial-and-peer-review-process#loc-peer-review-history)). If published, this will include your full peer review and any attached files.


If you choose “no”, your identity will remain anonymous but your review may still be made public.


**Do you want your identity to be public for this peer review?** For information about this choice, including consent withdrawal, please see our [Privacy Policy](https://www.plos.org/privacy-policy).

Reviewer #1: No

Reviewer #2: No

Reviewer #3: No

[NOTE: If reviewer comments were submitted as an attachment file, they will be attached to this email and accessible via the submission site. Please log into your account, locate the manuscript record, and check for the action link "View Attachments". If this link does not appear, there are no attachment files.]

While revising your submission, please upload your figure files to the Preflight Analysis and Conversion Engine (PACE) digital diagnostic tool, <https://pacev2.apexcovantage.com/>. PACE helps ensure that figures meet PLOS requirements. To use PACE, you must first register as a user. Registration is free. Then, login and navigate to the UPLOAD tab, where you will find detailed instructions on how to use the tool. If you encounter any issues or have any questions when using PACE, please email PLOS at [figures@plos.org](mailto:figures@plos.org). Please note that Supporting Information files do not need this step.

**Response:** Figures were evaluated with PACE and are confirmed to meet PLOS requirements.
